# Chinese ASCVD risk equations rather than pooled cohort equations are better to identify macro- and microcirculation abnormalities

**DOI:** 10.1186/s12872-020-01425-0

**Published:** 2020-03-24

**Authors:** Qiaowei Li, Fan Lin, Zhonghai Gao, Feng Huang, Pengli Zhu

**Affiliations:** 1 Department of Geriatric Medicine, Fujian Provincial Hospital, Fujian Provincial Center for Geriatrics, Fujian Provincial Key Laboratory of Geriatric Disease, Shengli Clinical Medical College of Fujian Medical University, 134 East Street, Fuzhou, 350001 Fujian China; 2 Department of ophthalmology, Fujian Provincial Hospital, Shengli Clinical Medical College of Fujian Medical University, Fuzhou, China

**Keywords:** ASCVD, Macrocirculation, Microcirculation

## Abstract

**Background:**

We hypothesized that discriminating the early subclinical organ damage would serve as a great opportunity for prevention against atherosclerotic cardiovascular disease (ASCVD). Brachial-ankle pulse wave velocity (baPWV), low retinal vascular fractal dimension, and albuminuria are surrogates of subclinical vascular changes.

**Methods:**

The aim of this study was to use Pooled Cohort Equations (PCE) and ASCVD risk equations derived from “Prediction for ASCVD Risk in China project (CHINA-PAR)” to observe the prevalence of macro- and microcirculation abnormalities. A total of 2166 subjects were involved. Characteristics were investigated using questionnaire and physical examinations. We calculated the urine albumin to creatinine ratio (UACR). The baPWV was measured using a fully automatic arteriosclerosis detector. The retinal vascular fractal dimension was measured by a semiautomated computer-based program. The 10-year ASCVD risk was estimated using the PCE and CHINA-PAR model.

**Results:**

The cut-off values for the elevated baPWV were 2.82 and 2.92% in the PCE model and CHINA-PAR model, respectively, with nearly 85% sensitivity and an average specificity of 74%. For low retinal fractal dimension, at the cut-off point of 3.8%, we acquired an acceptable sensitivity of 66.27–68.24% and specificity of 62.57–67.45%. All the C-statistics presented a significant improvement from the PCE model to the CHINA-PAR model (*P* < 0.05). For all categories—net reclassification improvement (NRI) values were significant and clearly varied (0.329, 0.183, and 0.104, respectively) depending on the cut-off set at 3%.

**Conclusion:**

Our study demonstrated that the CHINA-PAR equations rather than PCE could provide better identification of macro- and microcirculation abnormalities. A lower cut-off point for the subclinical vascular changes may be selected in a population from southeast China.

## Background

Cardiovascular disease (CVD) produces severe health and economic burden in China and globally [1, 2]. There were an estimated 93.8 million cases of CVD overall during 2016 in China, which is more than twice the cases that were reported in 1990 [1]. For better prevention of CVD and individualized therapy, several risk factors have been established. In 2013, the American College of Cardiology and the American Heart Association developed the Pooled Cohort Equations (PCE) to estimate the 10-year risk of developing a first hard atherosclerotic CVD (ASCVD) event (defined as the first occurrence of nonfatal myocardial infarction, coronary heart disease, death, or stroke) [3]. Due to ethnic differences, estimated risks may be over-estimated in Asian-American populations in the PCE model. A new tool to conduct ASCVD risk prediction called the Prediction for ASCVD Risk in China project (CHINA-PAR) was subsequently developed in 2016 [4]. The CHINA-PAR equations are based on traditional major risk factors with several new variables, validated by 4 Chinese cohorts, and aimed to serve as valuable predictor of ASCVD risk in the general Chinese population. However, studies have shown controversial results with the CHINA-PAR model. It outperformed PCE in ASCVD risk prediction in a rural northern Chinese population [5] but underestimated the risk in Mongolians [6]. Thus, further validation of ASCVD risk predictions in other Chinese populations needs to be performed.

ASCVD has a latency of many years and produces multiple subclinical manifestations during its progression. Therefore, we hypothesized that discriminating the early macro- and microvascular subclinical organ damage would provide a great opportunity to develop preventative interventions. In the macro-circulation, the measurement of arterial stiffness parameters was performed by brachial-ankle pulse wave velocity (baPWV) to predict the risk of future CV events and total mortality, which is recommended by the guidelines for the management of hypertension in Japan [7]. In the microcirculation, low retinal vascular fractal dimension indicates reduced complexity in the branching pattern of the microvasculature, independent of stroke risk factors [8], and albuminuria is associated with retinopathy, stroke, heart failure, and atherosclerosis [9–12].

Although these methods for early organ damage above are noninvasive, the technical complexity and time consumption may reduce the detection rate. Therefore, we aimed to use the ASCVD risk scores to observe the prevalence of macro- and microcirculation abnormalities in a Chinese population, achieving a more valuable equation by comparing the PCE and CHINA-PAR models.

## Methods

### Subjects

**Fig. 1:**
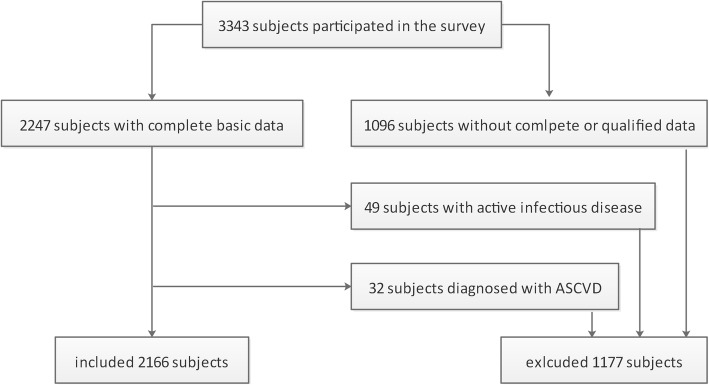
Flowchart of the study population. ASCVD, atherosclerotic cardiovascular disease

Our study was based on a cross-sectional study of hypertension conducted from July 2011 to November 2011. A total of 7 coastal villages in Fujian Province were randomly selected in cluster sampling. Voluntary participants aged 30 years or more living in the above selected areas for more than 5 years were enrolled. Subjects without complete data or qualified fundus photographs were excluded. We also excluded subjects previously diagnosed with ASCVD (including myocardial infarction, coronary heart disease and stroke) or with a suspicion of active inflammatory disease. Two thousand one hundred sixty-six subjects were involved finally (Fig. 1). This study was performed according to the World Medical Association Declaration of Helsinki and approved by the Ethics Committee of the Fujian Provincial Hospital. A written informed consent was obtained from all individuals.

### Data collection

Questionnaires were administered to collect following variables: age, smoking habits, alcohol consumption, medical history (including hypertension, diabetes, myocardial infarction, cardiomyopathy, congenital heart disease, peripheral vascular disease, congestive heart failure, stroke, acute and chronic inflammatory diseases, malignancy, and hematological disorders), and medications. Participants underwent physical examinations to measure height, weight, waist circumference, hip circumference, and blood pressure. Body mass index (BMI) was calculated as the weight (kg)/height^2^ (m^2^). Fasting blood samples were collected for laboratory testing including total cholesterol (TC), high-density lipoprotein cholesterol (HDL-C), low-density lipoprotein cholesterol (LDL-C), glycosylated hemoglobin (HbA1c), and serum creatinine. Urinary creatinine and albumin were measured from the morning urine sample. We calculated estimated glomerular filtration rate (eGFR) with the Chronic Kidney Disease Epidemiology Collaboration equation [13]. The urine albumin to creatinine ratio (UACR) was defined as the ratio of urine albumin to urine creatinine [14]. The subjects who had been diagnosed or already took related medication were defined as hypertensive [15]. Participants with a clear history, or were currently taking anti-diabetic medications, or with an HbA1c ≥6.5% were defined as diabetic [16].

### Measurement of baPWV

After resting for more than 10 min, the baPWV was measured using a fully automatic arteriosclerosis detector (Colin VP-1000 device; Colin Medical Technology Company, Komaki, Japan) by two professionals. We selected the right baPWV for analysis. The inter- and intra-observer variability coefficients were 0.93 (95% CI: 0.92–0.93) and 0.99 (95% CI: 0.98–0.99), respectively.

### Retinal fractal dimension

For documentation of non-mydriatic fundus photographs, two ophthalmologists used digital cameras (Topcon NW-8, TOPCON CORPORATION, Tokyo, Japan, and Nikon D90, Tokyo, Japan) centering on the optic disk with a capturing range of 45°. A semiautomated computer-based program (Singapore I Vessel Assessment version 3.0 software, jointly developed by Singapore National University and Singapore Eye Research Institute, Singapore) was used to measure retinal vascular fractal dimension in the range of 0.5–2 DD from the disc margin. In a random sample of 100 fundus photographs, we found no differences between intra-grader measurements. The intergrader correlation coefficient for Df was 0.90 (95% CI: 0.89–0.90).

### Assessment of ASCVD risk

The 10-year ASCVD risk was estimated using the PCE model recommended by the 2013 American College of Cardiology/American Heart Association (ACC/AHA) [3] and the CHINA-PAR model produced based on a Chinese population [4], respectively. The basic equation is $1-{{\mathrm{S}}_{10}}^{{\mathrm{e}}^{\left({\mathrm{IndX}}^{\prime}\mathrm{B}-{\mathrm{MeanX}}^{\prime}\mathrm{B}\right)}}$, in which S_10_ means baseline survival, lndX’B is calculated by sum of “Coeffcient × Value”, and MeanX’B stands for mean of “Coeffcient × Value” (details showed in Supplemental Table 1). The PCE included gender, age, race, diagnosis of diabetes, smoking, treated hypertension, systolic blood pressure, and lipid profile values (TC and HDL-C) as variables. The CHINA-PAR equations contained the major risk factors adopted in the PCE and additional variables including waist circumference, geographic region (northern vs. southern), urbanization (urban vs. rural), and family history of ASCVD.

### Definition of macro- and microcirculation abnormalities

The cut-off value for the normal and increased baPWV was 1400 cm/s [17]. The lowest quartile of the retinal vascular fractal dimension was defined as a low fractal dimension. Albuminuria was defined as UACR ≥30 mg/g [14].

### Statistical analysis

All statistical analyses were conducted using R version 3.5.3 and MedCalc® statistical software, and a *P*-value < 0.05 was considered statistically significant. Normally distributed continuous data are given as means ± SDs, skewed distributed continuous data are reported as median (25th, 75th percentiles), categorical variables are presented as the percentage of patients. Receiver operating characteristic (ROC) curves were applied to evaluate macro- and microcirculation abnormalities. Cut-off points were determined according to the Youden index with the best-combined sensitivity and specificity. Prognostic comparisons between models were done using Harrell’s concordance C-statistic. We explored categorical net reclassification improvement (NRI) to evaluate the stratification of effects from the PCE model to the CHINA-PAR model at the cut-off point derived from the ROC.

## Results

Table 1 shows characteristics of the study population. A total of 2166 individuals were enrolled in this study, with an average age of 51.91 ± 11.91 years and 37.4% men. The median UACR was 10.17 mg/g (interquartile range 4.13–22.98 mg/g). The mean retinal fractal dimension was 1.37 ± 0.05. The median baPWV for all subjects was 1346 cm/s, with an interquartile range of 1170–1600 cm/s. The prevalence of hypertension, diabetes mellitus, smoking, and drinking were 23.6, 8.5, 19.1, and 15.7%, respectively.

**Table 1:** Characteristics of study population

subjects, n	2166
age, years	51.91 ± 11.91
systolic BP, mmHg	126.89 ± 21.90
diastolic BP, mmHg	78.53 ± 11.84
WHR	0.85 ± 0.07
BMI, kg/m^2^	23.83 ± 3.42
total cholesterol, mmol/L	5.03 ± 1.05
HDL-C, mmol/L	1.23 ± 0.33
LDL-C, mmol/L	2.78 ± 0.88
eGFR, ml/min/1.73m^2^	103.25 ± 33.62
HbA1c, %	5.72 ± 0.68
UACR, mg/g	10.17(4.13, 22.98)
fractal dimension	1.37 ± 0.05
baPWV, cm/s	1346(1170, 1600)
male, n (%)	810(37.4)
hypertension history, n (%)	512(23.6)
diabetes history, n (%)	185(8.5)
smoking, n (%)	414(19.1)
drinking, n (%)	339(15.7)

Normally distributed continuous data are given as mean ± SD, skewed distributed continuous data are reported as median (25th, 75th percentiles), categorical variables are presented as a percentage of patients. *BP* Blood pressure, *WHR* Waist-hip rate, *BMI* Body mass index, *HDL-C* High density lipoprotein-cholesterol, *LDL-C* Low density lipoprotein-cholesterol, *eGFR* Estimated glomerular filtration rate, *HbA1c* Glycated haemoglobin A1c, *UACR* Urine albumin-to-creatinine ratio, *baPWV* Brachial-ankle pulse wave velocity

Cut-off values, sensitivity, and specificity with the highest Youden index are shown in Table 2. The cut-off values for the elevated baPWV were 2.82 and 2.92% in the PCE model and the CHINA-PAR model, respectively, with nearly 85% sensitivity and an average specificity of 74%. For the low retinal fractal dimension, at the cut-off point of 3.8%, we achieved an acceptable sensitivity of 66.27–68.24% and specificity of 62.57–67.45%. However, for albuminuria, neither the PCE model nor the CHINA-PAR model would achieve sensitivity higher than 61.14%. In this case, we set a new cut-off point at 3% for the high-risk category (PCE and CHINA-PAR models) for reclassification.

**Table 2:** Highest Youden index of macro- and microcirculation abnormalities and corresponding cut-off, sensitivity, and specificity

risk scores	Youden index	cut-off	sensitivity	specificity
elevated baPWV				
PCE	55.97	2.82	84.14	71.84
CHINA-PAR	62.18	2.92	84.25	77.93
low fractal dimension				
PCE	30.81	3.79	68.24	62.57
CHINA-PAR	33.72	3.77	66.27	67.45
albuminuria				
PCE	15.00	11.09	35.15	79.85
CHINA-PAR	18.52	3.06	61.14	57.38

Values are presented as %. *baPWV* Brachial-ankle pulse wave velocity, *PCE* Pooled cohort equation recommended by the 2013 American College of Cardiology and American Heart Association guidelines; CHINA-PAR, equations for 10-year ASCVD risk prediction in Chinese populations

**Fig. 2:**
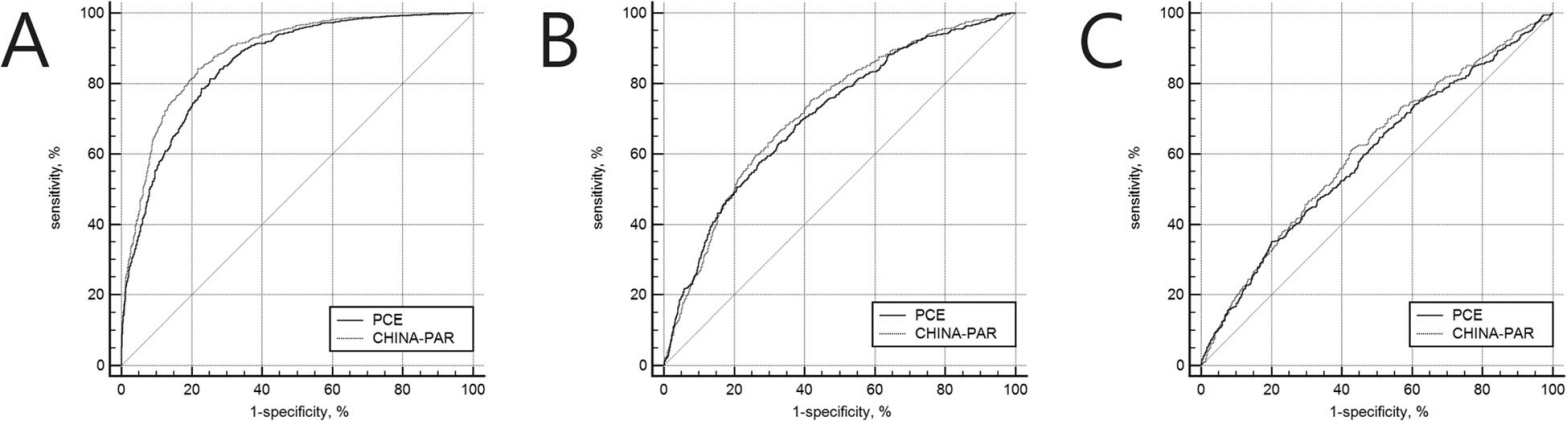
Receiver operating characteristic curves (ROC) for PCE and CHINA-PAR of macro- and microcirculation abnormalities. **a** ROC of elevated brachial-ankle pulse wave velocity (baPWV); **b** ROC of low fractal dimension; **c** ROC of albuminuria. PCE, pooled cohort equation recommended by the 2013 American College of Cardiology and American Heart Association guidelines; CHINA-PAR, equations for 10-year ASCVD risk prediction in Chinese populations

The area under the ROC curve calculated for discriminating elevated baPWV was 0.86 for PCE (95% CI: 0.84–0.87) and 0.88 for CHINA-PAR (95% CI: 0.87–0.90). To distinguish the low fractal dimension, the C-statistics were 0.71 (95% CI: 0.69–0.73) for PCE and 0.72 (95% CI: 0.70–0.74) for CHINA-PAR. The C-statistics to detect albuminuria were 0.59 (95% CI: 0.57–0.61) for PCE and 0.61 (95% CI: 0.59–0.63) for CHINA-PAR. All the C-statistics presented a significant improvement from the PCE model to the CHINA-PAR model (*P* < 0.05). All categorical-NRI values were significant and varied across groups (0.329, 0.183, and 0.104, respectively) depending on the cut-off set at 3%. (Table 3 and Fig. 2).

**Table 3:** Comparisons of PCE and CHINA-PAR with a difference in AUC and NRI for prediction of macro- and microcirculation abnormalities

	C-statistics of PCE model (95% CI)	C-statistics of CHINA-PAR model (95% CI)	∆AUC (95% CI)	*P*	categorical NRI (95% CI)	*P*
elevated baPWV	0.86 (0.84–0.87)	0.88(0.87–0.90)	0.028(0.020–0.036)	< 0.001	0.329(0.290, 0.368)	< 0.001
low fractal dimension	0.71 (0.69–0.73)	0.72 (0.70–0.74)	0.012 (0.002–0.022)	0.019	0.183(0.137, 0.228)	< 0.001
albuminuria	0.59 (0.57–0.61)	0.61 (0.59–0.63)	0.016 (0.005–0.027)	0.032	0.104(0.053, 0.156)	< 0.001

Cut-off for categorical NRI is 3.00%. PCE, pooled cohort equation recommended by the 2013 American College of Cardiology and American Heart Association guidelines; CHINA-PAR–equations for 10-year ASCVD risk prediction in Chinese populations; *AUC* Area under the receiver operative curve (C-statistics); *CI* Confidence interval, *NRI* Net reclassification improvement; *baPWV* brachial-ankle pulse wave velocity

## Discussion

To our knowledge, our study is the first to validate the incremental value of CHINA-PAR equations against PCE for discriminating macro- and microcirculation abnormalities.

In this cross-sectional study, we assessed the performance of the PCE and CHINA-PAR models for macro- and microcirculation abnormalities in a southern Chinese rural population.

Our results showed that both models showed acceptable discrimination of elevated baPWV and low retinal vascular fractal dimension. Compared to PCE, CHINA-PAR equations showed a significant improvement in the prevalence stratification of macro- and microcirculation abnormalities represented by elevated baPWV, low retinal vascular fractal dimension, and UACR. After resetting the cut-off for the high-risk group at 3%, the prevalence of the reclassification was significantly better in the CHINA-PAR equations than in the PCE.

Subclinical vascular changes both in macro- and microcirculation would progress in ASCVD.

As a representative marker of macro-circulation, arterial stiffness measured with the baPWV has the ability to predict future CV events and total mortality [7]. Although ageing exerts a strong influence on the baPWV [18], the reference values for the baPWV and Asian evidence showed 1400 cm/s is an appropriate cut-off [17, 19]. From the microcirculation perspective, retinal microvasculopathy may reflect small vessel disease. The lower fractal dimension which reflects a sparser retinal microvascular network is associated with stroke, Alzheimer’s disease, and more diffuse and severe coronary artery disease [20–23]. As demonstrated, albuminuria is significantly and independently associated with the presence and severity of atherosclerosis [12]. Therefore, we considered elevated baPWV, lower fractal dimension, and albuminuria proxies of macro- and microcirculation abnormalities.

Matthew et al. indicated that the PCE risk score provided a better estimate of racial differences in vascular function and structure than the Framingham risk score did [24]. Few former studies have assessed the association of ASCVD risk by CHINA-PAR equations with subclinical vascular changes. It may help to identify Chinese individuals at earlier time points who could benefit from early commencement or strengthening of medical treatment. The finding in the current study supports that the CHINA-PAR equations provides utility in identifying patients with elevated baPWV and low fractal dimension, in addition to a significantly improved risk assessment of all the macro- and microcirculation abnormalities as compared to PCE.

Since the CHINA-PAR equations were developed in 2016, the validations in real practice mostly focused on the northern population [5, 6]. To date, an approximately 6-fold difference in the total burden of CVD persists among provinces [1]; therefore, geographical strategies are needed to identify ASCVD throughout China in specific provinces. Our study proposed a lower cut-off point at 3% to classify individuals at high-risk for macro- and microcirculation abnormalities. The discrepancy may be attributed to the lowest rates for ASCVD in China being in the Fujian province and their dietary difference with a higher intake of proteins, unsaturated fatty acids, magnesium, zinc, and calcium. Our study also underscored that it is crucial to pay attention to those who belong to the low-risk group but as a result of the reclassification by the adjusted cut-off point, were then reclassified as high-risk, and therefore require more intense therapy.

Strengths of our study include three parameters to indicate subclinical vascular changes. We used the C-statistic and NRI to access and compare the effects of models. Our study also has a few limitations. The cross-sectional nature of the study would not clarify the causal relationship; therefore, a larger cohort in the future is required. A few subjects were not enrolled in the study because of noncompliance, leading to a possible selection bias. The use of a questionnaire is another limitation, which may cause recall bias. In addition, our results may not be generalized to other ethnicities, because the study was only validated in Chinese residents.

## Conclusion

Overall, our study demonstrated that the CHINA-PAR equations rather than PCE could provide better identification of macro- and microcirculation abnormalities. A lower cut-off point for subclinical vascular changes may be selected in a population from southeast China.

## Supplementary information

**Additional file 1: Supplemental Table 1**. comparison of CHINA-PAR and PCE models

## Data Availability

The datasets generated and analyzed during the current study are not publicly available due to the fact that the study is still followed up but are available from the corresponding author on reasonable request.

## Abbreviations

CVD: Cardiovascular disease
PCE: Pooled cohort equations
ASCVD: Atherosclerotic CVD
CHINA-PAR: Prediction for ASCVD risk in China project
baPWV: Brachial-ankle pulse wave velocity
TC: Total cholesterol
HDL-C: High-density lipoprotein cholesterol
LDL-C: Low-density lipoprotein cholesterol
HbA1c: Glycosylated hemoglobin
BMI: Body mass index
UACR: Urine albumin to creatinine ratio
eGFR: The estimated glomerular filtration rate
ROC: Receiver operating characteristic
NRI: Net reclassification improvement
