# Supplementary material for: Chinese ASCVD risk equations rather than pooled cohort equations are better to identify macro- and microcirculation abnormalities
Source: BMC Cardiovasc Disord. 2020 Mar 24;20:145. doi: 10.1186/s12872-020-01425-0 (PMC7092674; doi:10.1186/s12872-020-01425-0)
Supplement: Supplementary file 1 — Additional file 1: Supplemental Table 1. comparison of CHINA-PAR and PCE models [file 12872_2020_1425_MOESM1_ESM.docx]

| Supplemental Table 1. comparison of CHINA-PAR and PCE models | | | | |
| --- | --- | --- | --- | --- |
|  | CHINA-PAR Coefficients | | PCE Coefficients | |
| Variables | Men | Women | Men | Women |
| Ln(age),y | 31.97 | 24.87 | 12.344 | -29.799 |
| Ln(age),Squared | N/A | N/A | N/A | 4.884 |
| Ln(treated systolic BP),mmHg | 27.39 | 20.71 | 1.797 | 2.019 |
| Ln(untreated systolic BP),mmHg | 26.15 | 19.98 | 1.764 | 1.957 |
| Ln(total cholesterol),mg/dL | 0.62 | 0.06 | 11.853 | 13.54 |
| Ln(HDL-C),mg/dL | -0.69 | -0.22 | -7.99 | -13.578 |
| Ln(waist circumerence),cm | -0.71 | 1.48 | N/A | N/A |
| Current somker(1=Yes,0=No) | 3.96 | 0.49 | 7.837 | 7.574 |
| Diabetes(1=Yes,0=No) | 0.36 | 0.57 | 0.658 | 0.661 |
| Geographic region(1=Northern China,0=Southern China) | 0.48 | 0.54 | N/A | N/A |
| Urbanization(1=Urban,0=Rural) | -0.16 | N/A | N/A | N/A |
| Family history of ASCVD(1=Yes,0=No) | 6.22 | N/A | N/A | N/A |
| Ln(age)*Ln(treated systolic BP) | -6.02 | -4.53 | N/A | N/A |
| Ln(age)*Ln(untreated systolic BP) | -5.73 | -4.36 | N/A | N/A |
| Ln(age)*Current somker | -0.94 | N/A | -1.795 | -1.665 |
| Ln(age)*Family history of ASCVD | -1.53 | N/A | N/A | N/A |
| Ln(age)*Ln(total cholesterol) | N/A | N/A | -2.664 | -3.114 |
| Ln(age)*Ln(HDL-C) | N/A | N/A | 1.769 | 3.149 |
| Baseline survival | 0.97 | 0.99 | 0.91 | 0.97 |

PCE, pooled cohort equation recommended by 2013 American College of Cardiology and American Heart Association guidelines; CHINA-PAR, equations for 10-year ASCVD risk prediction in Chinese populations. Ln, natural logarithm; N/A, covariate was not included in the equation; BP, blood pressure; HDL-C, high-density lipoprotein cholesterol; ASCVD, atherosclerotic cardiovascular disease.
